# Establishing a consumer advisory group at the Australian Bragg Centre for Proton Therapy and Research

**DOI:** 10.1002/jmrs.746

**Published:** 2023-12-26

**Authors:** Melanie Penfold, Peter Gorayski, Julia Green, Kelly Skelton

**Affiliations:** ^1^ Australian Bragg Centre for Proton Therapy and Research South Australian Health and Medical Research Institute Adelaide South Australia Australia; ^2^ Department of Radiation Oncology Royal Adelaide Hospital Adelaide South Australia Australia; ^3^ Department of Allied Health and Human Performance University of South Australia Adelaide South Australia Australia; ^4^ University of South Australia Adelaide South Australia Australia

## Abstract

The Australian Bragg Centre for Proton Therapy and Research (ABCPTR) established the Bragg Consumer Advisory Group (BCAG) in 2023. The ABCPTR, being the first of its kind in Australia, will offer proton therapy treatment for challenging solid tumours with the potential to reduce radiation‐induced side effects. With over 110 Proton Beam Therapy (PBT) centres globally, Australian patients currently can apply to access government funded treatment overseas, however, international travel for treatment presents various, significant challenges. Consumer engagement in healthcare plays a pivotal role in navigating the multifaceted journey of cancer treatment and can complement cancer control strategies by ensuring the practicalities of the cancer journey are realised. The ABCPTR aims to involve consumers in decision‐making processes, especially as it prepares to open Australia's first national proton therapy centre. The aim of this commentary is to highlight the importance of involving consumers in cancer care, and to demonstrate how this was done in Australia's first proton therapy centre. To establish a consumer engagement team, ABCPTR utilised existing clinical staff. The team's formation and upskilling were integral to the project's success. The engagement framework was developed based on the five stages of commitment by the Australian Health Research Alliance and Western Australian Health Translation Network. The ABCPTR consumer engagement team successfully created a community engagement framework and upskilled in consumer engagement principles over 9–12 months. An Expression of Interest (EOI) was launched, resulting in the formation of the BCAG comprising of 10 members with diverse backgrounds and experiences. The BCAG has been actively involved in decision‐making processes, with a consumer‐led chair and co‐chair in place. The group's feedback is expected to influence key performance indicators for the centre. The establishment of the BCAG at the ABCPTR emphasises the importance of integrating patient and community perspectives into clinical initiatives. This proactive approach ensures that processes remain patient‐centred. The ongoing multi‐level consumer engagement strategy aims to shape a more inclusive approach to cancer care in Australia, especially concerning PBT.

## Graphical abstract

Consumer engagement in healthcare plays a pivotal role, particularly in navigating the multifaceted journey of cancer treatment and can complement cancer control strategies by ensuring the practicalities of the cancer journey are realised. The Australian Bragg Centre for Proton Therapy and Research (ABCPTR) established the Bragg Consumer Advisory Group (BCAG) in 2023.

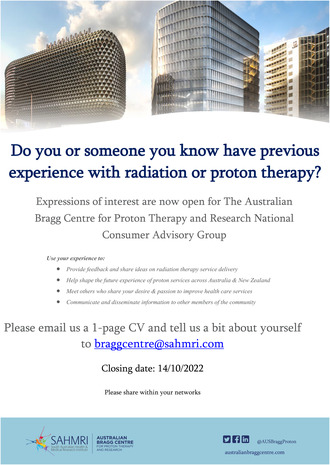

## Introduction

In 2023 the Australian Bragg Centre for Proton Therapy and Research (ABCPTR) embarked on creating a consumer engagement team with the primary goal to establish a consumer advisory group to advise on proton beam therapy from a patient and community perspective. Consumer engagement in healthcare is widely accepted as valuable and is linked with improvements in both quality and safety of healthcare services.^1^ This, in part, is due to the unique and diverse perspectives of our community and consumers who use these services. The cancer treatment journey is often complex and lengthy, with multiple medical and allied health professionals required to work in unison to ensure optimal outcomes for patients, this is especially true for the ABCPTR who will be servicing the national population. The ABCPTR is committed to engaging, involving and collaborating with consumers to facilitate continuous improvement, ensuring optimal treatment outcomes for people affected by cancer. To this end, the Bragg Consumer Advisory Group (BCAG) is actively participating in decision‐making processes in the lead up to the clinical opening of Australia's first national proton therapy centre. The term ‘consumers’ refers broadly to patients, a person caring for a loved one with cancer, or a family member or friend.^2^

## Background

The ABCPTR, currently under construction, will soon provide an additional treatment option to suitable patients with otherwise difficult to treat solid tumours. The first of its kind in Australia, the synchrotron and delivery system at the ABCPTR can deliver precise doses of radiation to the target volume while sparing normal tissue, compared with conventional x‐ray therapy. The reduction in low doses of radiation to normal tissue has the potential to minimise radiation‐induced side effects, translating to an increased quality of life for long‐term survivors and reduced burden on the healthcare system. This is particularly beneficial for the treatment of children and adolescents and young adults (AYA) where the risk of developing long‐term complications from dose to normal tissue is much higher. The ABCPTR will, therefore, treat a significant proportion of patients 25 years and under.

Globally, there are over 110 proton beam therapy (PBT) centres in operation with the majority located in the USA, Europe and Asia.^3^ Currently, in Australia, patients who might benefit from PBT are required to apply to the federal government funding scheme Medical Treatment Overseas Program (MTOP) to access PBT overseas.^4^ The decision to support, or not, patient access to PBT overseas is made on a case‐by‐case basis.

Significant obstacles are presented to patients and families wishing to pursue treatment overseas including potential time delays to treatment, coordination of adjuvant treatments, long travel distances and spending time away from family and support networks.

The Medical Services Advisory Committee (MSAC) has approved specific tumour indications to be bulk‐billed for PBT once treatment is available within Australia.^5^ The ABCPTR will service the eligible national population creating unique challenges for both ABCPTR and patients. These challenges, such as travelling for treatment and residing away from home for interstate patients, are not too dissimilar to the challenges currently faced by Australians travelling to access PBT overseas.

## The ABCPTR Consumer Engagement Team

At the ABCPTR, a limited number of core clinical and research project staff have been appointed in the lead up to clinical service provision, including project managers, radiation oncologists, medical physicists, radiation therapists and registry staff. In the absence of dedicated personnel with prior experience or specialised knowledge in consumer engagement the decision was made to establish consumer engagement activities from the ground up with existing clinical staff. The ABCPTR consumer engagement team, therefore, consists of a radiation oncologist, a clinical implementation project manager, two radiation therapists and a registry project manager.

## The ABCPTR Consumer Engagement Framework

Consumers can be engaged or involved within an organisation at varying levels. ABCPTR used the levels of participation, as defined by Cancer Australia, to create a strategy for engaging consumers at each of the levels including informing, consulting, involving, partnership and consumer‐led.^6^ Noting that there is a broad spectrum of engagement or involvement, the team commenced with the ‘lower’ level of engagement, with the view of reaching consumer‐led engagement within 2–3 years.

**Figure 1:**
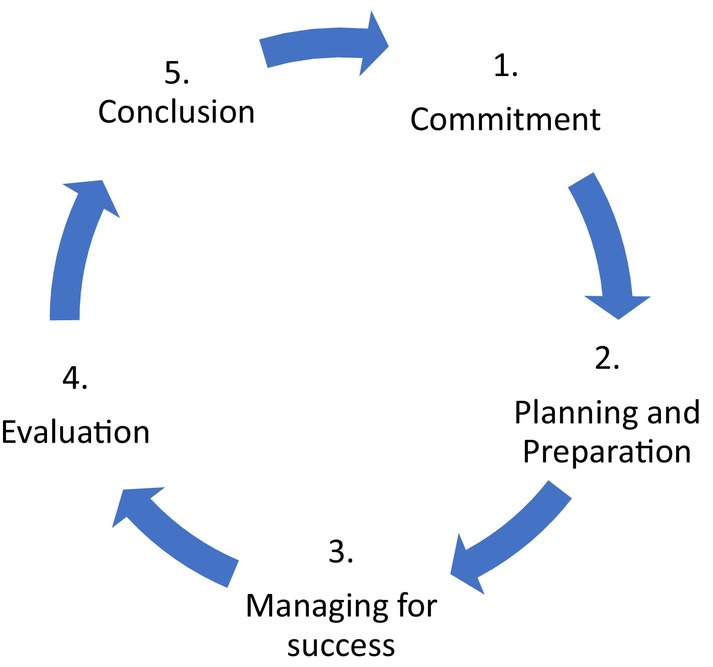
The five stages of consumer engagement.

The ABCPTR team developed a consumer engagement framework using the five stages of commitment produced by the Australian Health Research Alliance and Western Australian Health Translation Network.^7^ The five stages include 1. Commitment, 2. Planning and preparation, 3. Managing for success, 4. Evaluation and 5. Conclusion (Fig. 1).^7^

The first step, as part of the commitment stage was to upskill the team in the principles and practices of consumer engagement. This took 9–12 months and included activities such as attending South Australian Health and Medical Research Institute (SAHMRI) Consumer Engagement Community of Practice webinars, engaging with local experts, reading books and articles and additional self‐directed learning. Many online resources and tool kits were utilised, including the National Framework for Consumer Involvement in Cancer Control, produced by Cancer Voices Australia and the Australian Government.^2^ Regular meetings were held by the ABCPTR consumer engagement team to discuss what we had learnt and our next steps in facilitating consumer engagement activities. In particular, the advice sought from local consumer engagement experts at SAHMRI with regards to establishing a consumer advisory group was enormously valuable.

In addition to the growing evidence supporting consumer engagement, the ABCPTR will be mandated to implement the National Health and Safety Quality Service (NHSQS) Standards once clinically operational. The primary aims of the NHSQS is to provide key performance indicators to improve the quality of health service provision and to protect consumers from harm. Eight standards are assessed, including *Partnering with Consumers Standard*, which details the leaders of health service organisations to develop, implement and maintain systems to partner with consumers with respect to planning, design, delivery, measurement and evaluation of care.^8^

In stage 2, the ABCPTR consumer engagement team produced a statement of principles, highlighting our mission and vision for the ABCPTR and additionally, a position statement detailing our commitment to engaging with consumers. Finally, the team developed an advisory group proposal including costings for approval by the ABCPTR Board. Members of the BCAG are remunerated for their time and expenses according to SA Health Policy.^9^ An Expression of Interest (EOI) flyer was created to target those in the community with lived experience of radiation or proton therapy, including family and carers (see Appendix S1). In particular we were looking for recent experience of PBT in an overseas centre from the perspective of an adult, AYA and paediatric patient. As the ABCPTR is a national service, membership was sought from all states and territories in Australia. The EOI was emailed to various national cancer charities and non‐government organisations (NGO) such as Canteen and cancer departments around Australia for dissemination to their patients and community. Interested individuals were asked to provide a summary about themselves and a 1‐page CV.

## Key Milestones

Numerous EOIs were received, and the ABCPTR consumer engagement team embarked on a selection process. Members of the BCAG were selected based on their lived experience of PBT or radiation therapy, their role (family, carer, or patient), age, location, background and their skill set. The ABCPTR consumer engagement team set aside 2 hours to review all EOIs and as a group, make a selection based on the factors mentioned. Ten members sit on the BCAG with nine of these members either a patient or family member of a patient and one consumer advisory advocate from a national NGO. Membership includes first nations and culturally and linguistically diverse individuals.

An in‐person induction was held at the beginning of 2023 at SAHMRI, Adelaide and all consumers were supported to attend with flights and accommodation provided. Bringing the group together for the first face‐to‐face meeting was hugely beneficial in forming and strengthening relationships.^10^ The BCAG were invited to tour the ABCPTR while under construction and were able to walk through the treatment areas and experience firsthand, the patient pathway from treatment planning to delivery including standing in the vast cavities of the bunker rooms prior to equipment installation. Witnessing the centre at this unique phase of construction provided the group with an opportunity to visualise the service pre‐opening and provide comments on design, patient flow and experience.

**Figure 2:**
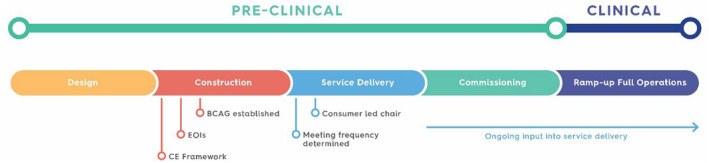
Project timeline including consumer engagement (CE) framework, expressions of interest (EOI) and Bragg Consumer Advisory Group (BCAG) established within the pre‐clinical phase of the Australian Bragg Centre for Proton Therapy and Research project.

The ABCPTR consumer engagement team is currently in the third stage of the framework and is actively working with the BCAG to ensure involvement in all aspects of current project work. A consumer‐led chair and co‐chair have been selected, a pivotal step towards fostering effective collaboration and cohesion among the national consumers and ensuring representation of the collective consumer group, by the consumer group. It is especially pertinent to note that engaging with the BCAG team early during the pre‐clinical stage is advantageous in setting up policies, procedures and pathways that benefit patients from the outset (Fig. 2). This includes gathering feedback from consumers with firsthand MTOP experience, not only from a treatment perspective, but also the psychological and social support requirements for patients and families to bolster their overall wellbeing.

## Measuring Success

The development of the consumer advisory group aligns with the growing body of evidence supporting consumer engagement in healthcare and fulfils requirements as set out by the NSQHS. Presently, the chair of the BCAG reports to the ABCPTR Board bi‐annually, enabling reciprocal updates and feedback. Future goals include integrating the advisory group into the centre's governance structure and cultivating consumer‐led engagement. Measures of success will involve evaluating the advisory group's impact on service provision and patient outcomes, tracking participation levels and continuously seeking feedback for further improvement. Anonymous feedback forms will be sent to the group at the end of each year and at additional times as required. Feedback received from the BCAG will be included in key performance indicators for the centre including improved patient satisfaction and enhanced access to wellbeing services for patients and their families. Measured success should be described by the level of engagement and collaboration achieved with the BCAG by the ABCPTR and ensuring their perspectives are effectively incorporated into decision‐making for the centre.

## Conclusion

This commentary underscores the creation and implementation of the BCAG at the ABCPTR. The goal is to enrich the centre's decision‐making process by harnessing lived experiences and unique insights of the cancer patient community, their carers and the wider community. This initiative embodies the larger philosophy of a patient‐centred approach in healthcare, significantly embraced within the field of radiation oncology and particularly relevant to PBT.

This commentary also details the formation and upskilling of the ABCPTR consumer engagement team utilising existing ABCPTR clinical staff members, highlighting that consumer engagement strategies can be achieved on limited resourcing. As an evolving entity, the team is striving to progress from ‘informing’ to ‘consumer‐led’ engagement, thereby encapsulating all levels of participation. This evolutionary approach lends flexibility and adaptability to the process, enhancing consumer engagement over time. The formation of the consumer advisory group at the ABCPTR is an example of proactively integrating patient and community perspectives into clinical and research initiatives, prior to clinical opening, to ensure that processes and policies are centred on the patient in the first instance. Once open, it is hoped that this ongoing, multi‐level consumer engagement strategy will minimise challenges associated with receiving PBT at the ABCPTR by shaping a more inclusive and holistic approach to cancer care in the Australian context.

## Conflict of Interest

The authors declare no conflict of interest.

## Supporting information


**Appendix S1.** Supplementary material.

## Data Availability

Data sharing is not applicable to this article as no new data were created or analyzed in this study.

## References

[1] Johnson A , Silburn K . Community and consumer participation in Australian health services‐An overview of organisational commitment and participation processes. Aust Health Rev 2000; 23: 113–121. 10.1071/ah000113a 11186043

[2] Cancer Australia, Cancer Voices Australia . National Framework for Consumer Involvement in Cancer Control. Cancer Australia, 2011. 50 p.

[3] Particle Therapy Cooperative Group, 2023 [cited 2023 August 10]. Available from: https://www.ptcog.site/.

[4] Department of Health and Aged Care . Medical Treatment Overseas Program, 2023 [cited 2023 May 23]. Available from: https://www.health.gov.au/our‐work/medical‐treatment‐overseas‐program.

[5] Department of Health and Aged Care . Medical Services Advisory Committee, 2021 [cited 2023 June 2]. Available from: http://www.msac.gov.au/internet/msac/publishing.nsf/Content/1638‐public.

[6] Cancer Australia . Consumer Involvement Toolkit, 2023 [cited 2023 June 4]. Available from: https://consumerinvolvement.canceraustralia.gov.au/.

[7] Australian Health Research Alliance, Western Australian Health Translation Network . Involving Consumers in Health and Medical Research, 2021 [cited 2023 June 4]. Available from: https://wahtn.org/platforms/consumer‐and‐community‐involvement/cci‐handbook/.

[8] ACSQHC . Australian Commission on Safety and Quality in Health Care, 2023 [cited 2023 June 4]. Available from: https://www.safetyandquality.gov.au/standards/nsqhs‐standards/partnering‐consumers‐standard.

[9] SA Health . Sitting Fees and Reimbursement for External Individuals Policy, 2021.

[10] Blanchard A , McBride A . Putting the group in “group” in group meetings: Entitativity in face‐to‐face and online meetings. In: Meinecke AL , Allen JA , Lehmann‐Willenbrock N (eds). Managing Meetings in Organisations, Vol 20. Emerald Publishing Limited, Bingley, 2020; 71–92.

