# Supplementary material for: Establishing a consumer advisory group at the Australian Bragg Centre for Proton Therapy and Research
Source: J Med Radiat Sci. 2023 Dec 26;71(Suppl 2):77–81. doi: 10.1002/jmrs.746 (PMC11011579; doi:10.1002/jmrs.746)
Supplement: Supplementary file 1 — Appendix S1. Supplementary material. [file JMRS-71-77-s001.pdf]

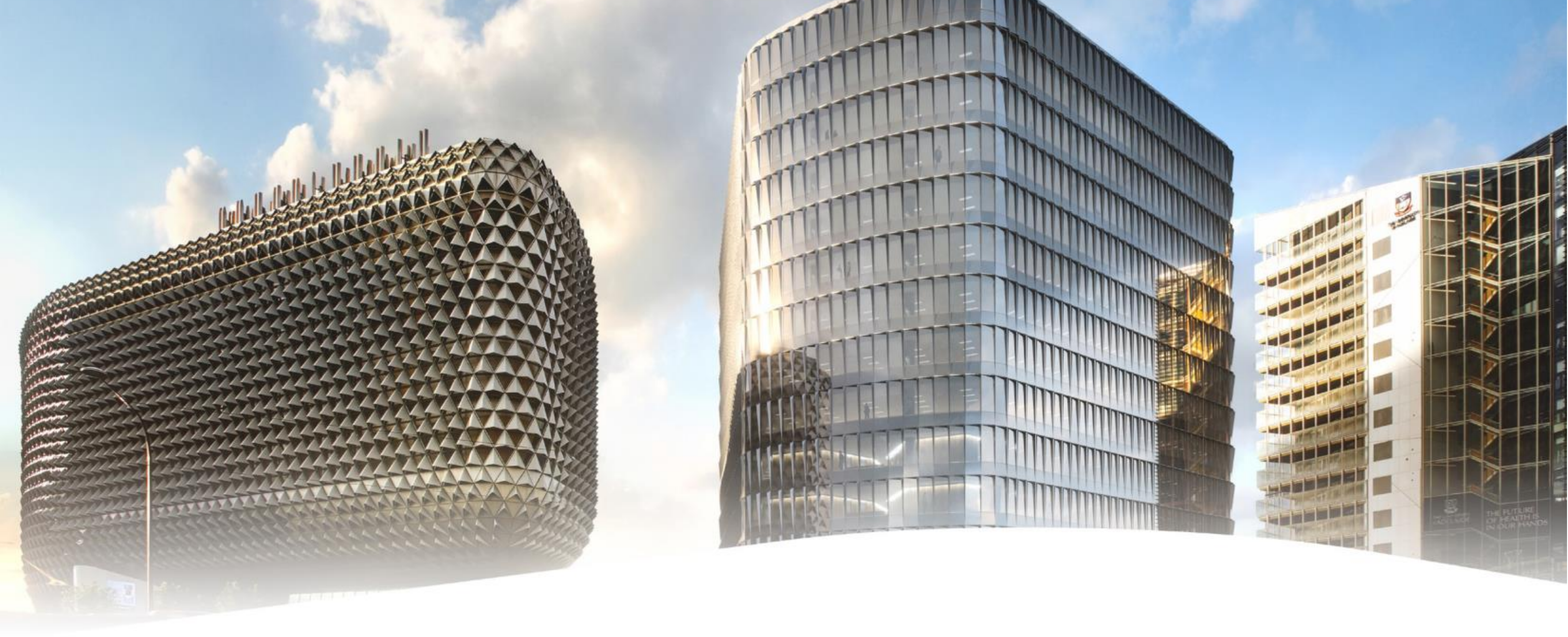

Do you or someone you know have previous experience with radiation or proton therapy?

Expressions of interest are now open for The Australian Bragg Centre for Proton Therapy and Research National Consumer Advisory Group

Use your experience to:

- *Provide feedback and share ideas on radiation therapy service delivery*
- *Help shape the future experience of proton services across Australia & New Zealand*
- *Meet others who share your desire & passion to improve health care services*
- *Communicate and disseminate information to other members of the community*

Please email us a 1-page CV and tell us a bit about yourself to braggcentre@sahmri.com

Closing date: 14/10/2022

Please share within your networks

SAHMRI South Australian Health & Medical Research Institute
AUSTRALIAN BRAGG CENTRE FOR PROTON THERAPY AND RESEARCH

@AUSBraggProton
australianbraggcentre.com
